# Supplementary figures and images for: Adenophora triphylla var. japonica Inhibits Candida Biofilm Formation, Increases Susceptibility to Antifungal Agents and Reduces Infection
Source: Int J Mol Sci. 2021 Nov 21;22(22):12523. doi: 10.3390/ijms222212523 (PMC8624521; doi:10.3390/ijms222212523)

Figure S1.

(a)

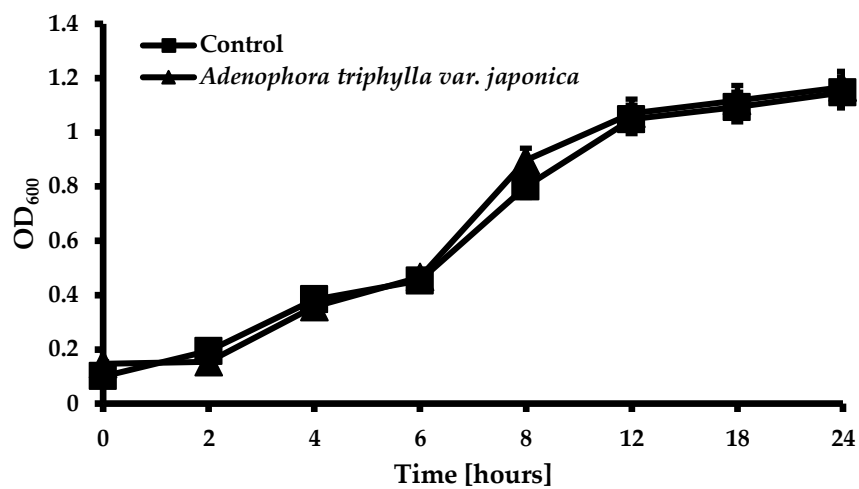

(b)

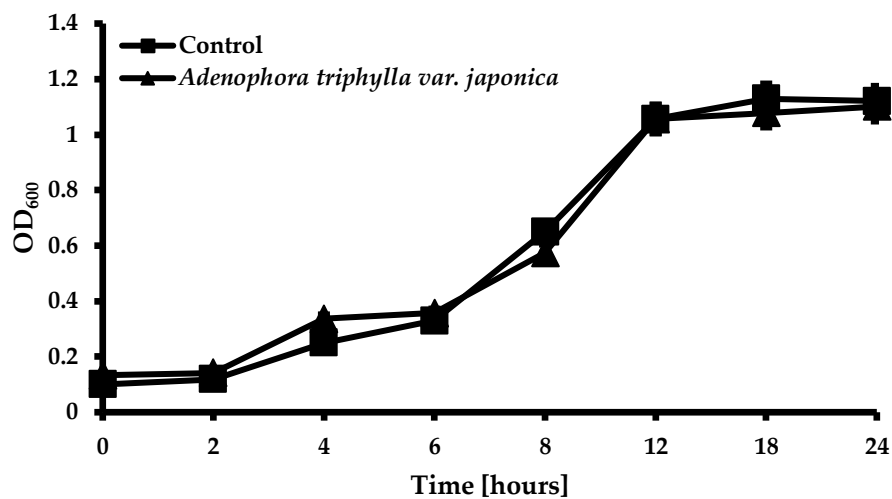

(c)

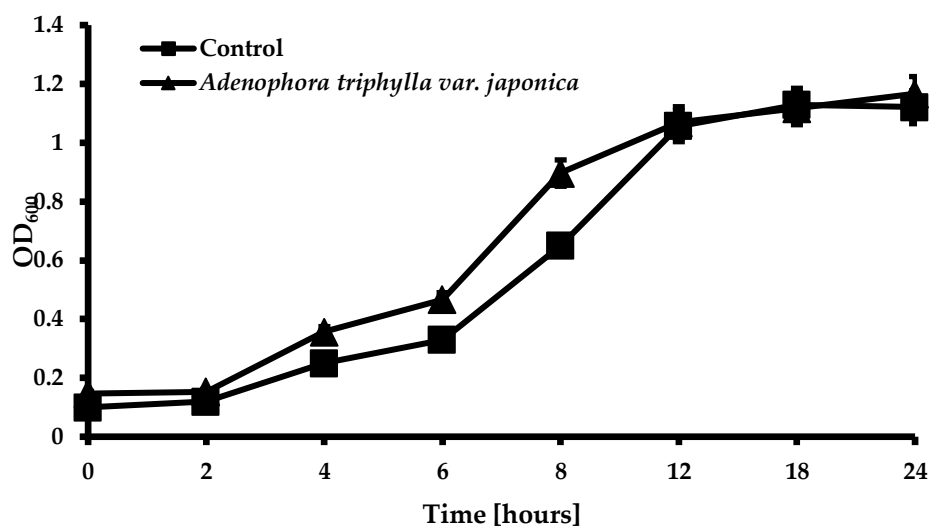

(d)

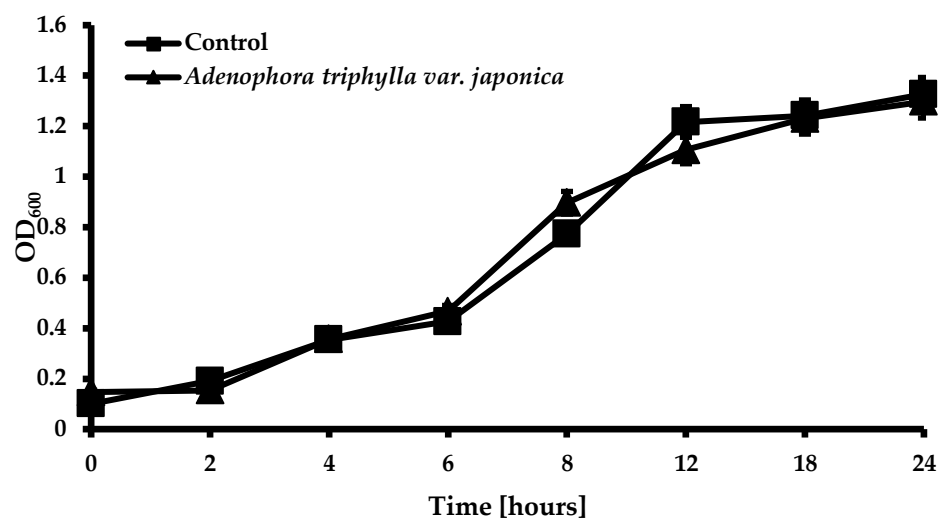

(e)

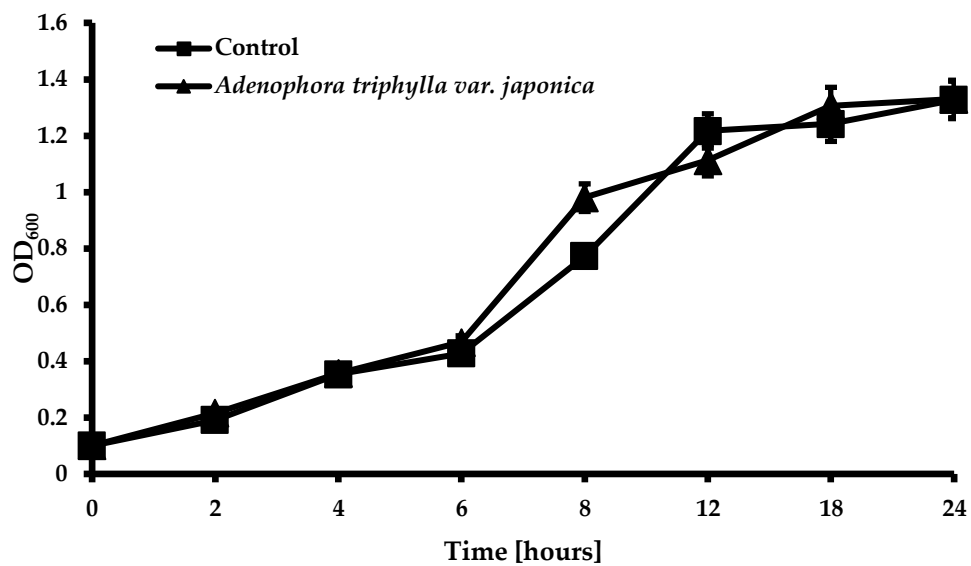

Supplement: Supplementary file 1 [file ijms-22-12523-s001.zip › ijms-1419792-supplementary.pdf]
